# Computed tomography radiomics for the prediction of thymic epithelial tumor histology, TNM stage and myasthenia gravis

**DOI:** 10.1371/journal.pone.0261401

**Published:** 2021-12-20

**Authors:** Christian Blüthgen, Miriam Patella, André Euler, Bettina Baessler, Katharina Martini, Jochen von Spiczak, Didier Schneiter, Isabelle Opitz, Thomas Frauenfelder

**Affiliations:** 1 Institute of Diagnostic and Interventional Radiology, University Hospital of Zurich, Zurich, Switzerland; 2 Department of Thoracic Surgery, University Hospital of Zurich, Zurich, Switzerland; University of Jordan, JORDAN

## Abstract

**Objectives:**

To evaluate CT-derived radiomics for machine learning-based classification of thymic epithelial tumor (TET) stage (TNM classification), histology (WHO classification) and the presence of myasthenia gravis (MG).

**Methods:**

Patients with histologically confirmed TET in the years 2000–2018 were retrospectively included, excluding patients with incompatible imaging or other tumors. CT scans were reformatted uniformly, gray values were normalized and discretized. Tumors were segmented manually; 15 scans were re-segmented after 2 weeks by two readers. 1316 radiomic features were calculated (pyRadiomics). Features with low intra-/inter-reader agreement (ICC<0.75) were excluded. Repeated nested cross-validation was used for feature selection (Boruta algorithm), model training, and evaluation (out-of-fold predictions). Shapley additive explanation (SHAP) values were calculated to assess feature importance.

**Results:**

105 patients undergoing surgery for TET were identified. After applying exclusion criteria, 62 patients (28 female; mean age, 57±14 years; range, 22–82 years) with 34 low-risk TET (LRT; WHO types A/AB/B1), 28 high-risk TET (HRT; WHO B2/B3/C) in early stage (49, TNM stage I-II) or advanced stage (13, TNM III-IV) were included. 14(23%) of the patients had MG. 334(25%) features were excluded after intra-/inter-reader analysis. Discriminatory performance of the random forest classifiers was good for histology(AUC, 87.6%; 95% confidence interval, 76.3–94.3) and TNM stage(AUC, 83.8%; 95%CI, 66.9–93.4) but poor for the prediction of MG (AUC, 63.9%; 95%CI, 44.8–79.5).

**Conclusions:**

CT-derived radiomic features may be a useful imaging biomarker for TET histology and TNM stage.

## Introduction

Thymic epithelial tumors (TET) are the most common primary tumor of the anterior mediastinum in adults and include thymomas, thymic carcinomas (TC) and thymic neuroendocrine tumors. While the metastatic tendency is low, all TET can show infiltrative growth, most commonly affecting the mediastinal pleura and the pericardium [1].

Resection status and tumor stage are the most important prognostic factors in thymomas and TCs [2]. In early stages (TNM stages I-II), thymomas are primarily resected, and achieve long, recurrence-free survival without adjuvant therapy, while advanced-stage TETs (TNM stages III-IV) require an interdisciplinary, multimodality approach comprised of an individual selection of induction chemotherapy, radical resection, adjuvant chemotherapy and sometimes radiotherapy [2]. Computed tomography (CT) and magnetic resonance imaging (MRI) are pivotal in the diagnostic workup of TETs, and preoperative biopsy may be avoided in resectable tumors. However, the available imaging modalities are often not able to reliably detect early stages of infiltration into adjacent structures [3].

The histologic classification of the composition of cell types in thymomas proposed by the World Health Organization (WHO) is another independent prognostic factor [4]. Qualitative imaging features such as smooth tumor margins or a round shape have been shown to correlate with WHO type A or B tumors, as opposed to irregular margins or TETs with necrotic components, which are more frequently encountered in WHO type C tumors [5]. However, due to a considerable overlap of imaging features, a confident visual classification is difficult and prone to subjectivity [5,6].

TET are strongly associated with the paraneoplastic disease myasthenia gravis (MG). Patients with MG suffer from various degrees of symptoms related to muscle weakness, and are at risk of developing severe complications, including myasthenic crisis, a possibly life-threatening postoperative condition after thymomectomy. This implies specialized perioperative care [7], and it is important to screen patients with TET for the presence of MG prior to surgical resection [8], however, the diagnostic workup of MG is complex [9].

A possible remedy to these problems could be the extraction of additional quantitative information from imaging data. For this field of study, the term “radiomics” was coined, as an addition to the related areas of genomics, proteomics, and metabolomics. Radiomics is based on deriving mathematically defined features from images, with the aim to detect and quantify information at levels surpassing the human visual system [10].

Machine learning classifiers trained on CT-based radiomic features as input have previously shown promising performance in characterizing lesions from various tissues [11]. In the case of TETs, studies have evaluated the use of logistic regression models for the classification of histologic type [12–18] and stage [13,17,19]. Only one study was identified that evaluated machine learning classifiers trained on CT-based radiomic features to predict the histologic type [15]. Additionally, most previous studies used the well-established Masaoka-Koga system [20], while the more recent 8^th^ edition TNM staging system for TET, might be able to improve stratification [8]. Predicting the TNM stage from CT-based radiomics has been researched by two groups employing logistic regression models [17,18], however, for the use of machine learning classifiers (e.g., Random Forest) in the setting of TET, published literature is currently limited to MRI-based radiomics [21].

This work strived to test the classification performance of a random forest classifier trained on radiomic features derived from three-dimensional volumes of interest (VOI) of contrast-enhanced CT scans for three categories: 1) histologic type, 2) TNM tumor stage and 3) the presence of preoperative myasthenia gravis (MG).

## Materials and methods

This retrospective study was approved by the local ethics committee (KEK Zürich, cantonal ethics committee Zurich, Switzerland), and the need for informed consent was waived. Patients undergoing surgery for TET in the years 2000–2018 were identified and included according to the following criteria: 1) patient age of 18 or older 2) no history of prior resection for TET or other neoplasms 3) no previous radio-/chemotherapy. From the acquired list, patients were excluded according to the following criteria: 1) incompatible or lacking CT scan, 2) unenhanced CT scan only or 3) anterior mediastinal tumors other than thymomas or thymic carcinoma.

TETs were divided into low-risk TETs (LRT, WHO types A, AB, B1) and high-risk TETs (HRT, WHO types B2-3,C) as previously proposed [10], as well as in early stage lesions (TNM I-II) and advanced stage lesions (TNM III-IV), following the implications for therapy, prognosis and survival [2,14]. The standard of reference was the surgical report and the report of the histopathologic workup of the resected TETs.

### Image acquisition and preprocessing

Due to the long inclusion range, CT scans were acquired using a variety of scanners: Siemens (Siemens Healthineers, Forchheim, Germany; SOMATOM Definition series (n = 36), SOMATOM Force (n = 6), SOMATOM Sensation series (n = 2), SOMATOM Biograph Series (n = 2), SOMATOM Edge Plus (n = 1)), GE (GE Healthcare Systems, Chicago, IL, USA; Lightspeed Series (n = 5), Discovery Series (n = 3), BrightSpeed (n = 1), Optima CT660 (n = 1)), Philips (Philips Healthcare, Amsterdam, NL; model iCT series (n = 2), Mx8000 IDT 16 (n = 1), Brilliance 40 (n = 1)) and Canon (Canon Medical Systems, Volketswil, CH; model Aquilion (n = 1)). The scanning parameters were: median reference tube voltage, 120 kVp (range 90–140 kVp), median exposure 195 mAs (IQR 122–265 mAs), matrix size 512 × 512, median slice thickness 2 mm (IQR 2–2.5 mm), reconstructed with available soft tissue convolutional kernels. All included CT studies were contrast-enhanced, using a routine chest CT protocol with arterio-venous phase (delay: 25–30 s).

Contrast-enhanced computed tomography (CT) scans were de-identified using the built-in full anonymization functionality of the PACS viewer (Syngo.via, Siemens Healthineers, Forchheim, Germany). Because some texture features require identical spatial resolution to be comparable, the image slices were reformatted to a uniform in-plane resolution of 1x1 mm^2^ and slice thickness of 2 mm, using a custom MATLAB script (MathWorks, Inc., Natick, MA, USA).

### Radiologic assessment

All tumors were evaluated by two radiologists (with 5 and 7 years of experience, respectively) blinded to the results of the histopathologic and surgical workup. The tumor stage was assessed as “early” or “advanced” according to the abovementioned criteria based on the 8^th^ edition TNM classification. Contact of the tumor to structures without a visible separating layer of mediastinal fat was counted as possible infiltration. Radiologists were also asked to categorize tumors as appearing “high risk” or “low risk” based on morphological features described in the literature: Round, smoothly contoured, homogenously enhancing lesions were counted as “low risk”, whereas irregular or lobulated, heterogenous lesions with possible necroses were counted as “high risk” [5,6].

### Segmentation

3D volumes of interest (VOI) including the whole tumor were segmented manually (axial view, slice by slice), using the software 3D-Slicer (http://slicer.org/, version 4.10.2). The segmentation time for a single tumor varied by tumor size and was in the range between five and ten minutes. As differences in segmentation may influence calculated features, segmentation was repeated by the original reader for a random subset of 15 CT scans after two weeks, to assess intra-observer variability. The same subset was also segmented by a second radiologist to assess inter-observer variability.

### Feature extraction

To account for technical differences, gray values were normalized to mean and standard deviation, removing outliers greater than 3 standard deviations [22]. Default values were used for all extraction settings, including a fixed bin width of 25 for gray value discretization. A total of 1316 radiomic features (i.e., filter-feature combinations) were extracted from original and filtered images (Laplace of Gaussian filter with parameter σ set to 1–5 (a higher value emphasizing on coarser textures), wavelet filter with all combinations of high-pass (H) and low-pass (L) filters), using the library *pyRadiomics* (version 2.1.2) [23]. The computation of all features took less than ten seconds per case. Features were based on first-order statistics, shape, gray level co-occurrence matrix (GLCM), gray level run length matrix (GLRLM), gray level size zone matrix (GLSZM), neighboring gray tone difference matrix (NGTDM) and gray level dependence matrix (GLDM) as described at http://pyradiomics.readthedocs.io (accessed 05.01.2021); the mathematical feature definitions are consistent with the Image Biomarker Standardization Initiative [24].

### Feature selection and model evaluation

Due to a high number of features and the susceptibility of ML models to overfit, feature selection was necessary. First, the intraclass correlation coefficient (ICC) of each feature was calculated in a two-way mixed, single measure approach for consistency (ICC(3,1)), and features with less than good inter- or intrareader agreement (ICC(3,1)<0.75) were excluded.

**Fig 1:**
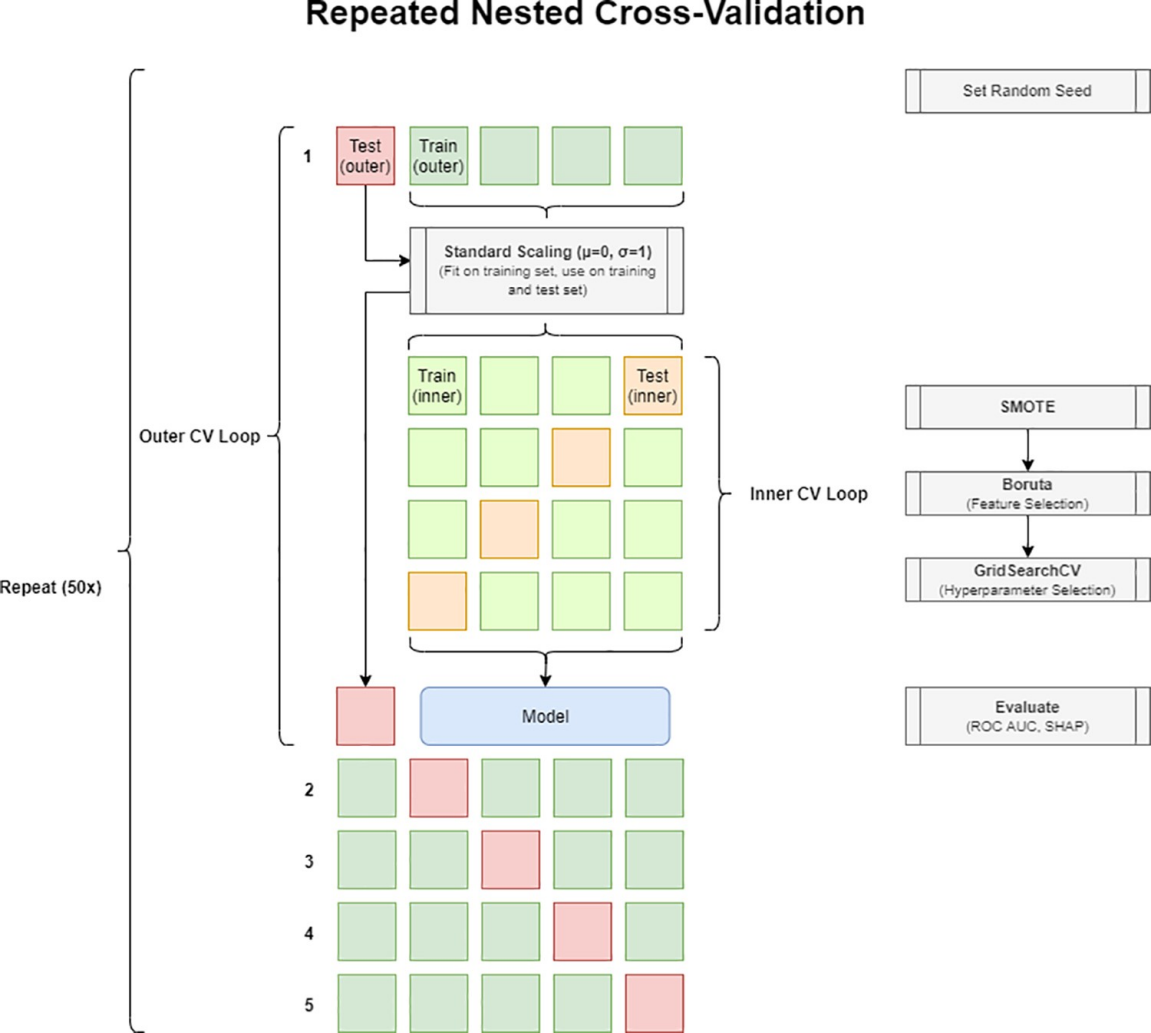
Repeated nested cross-validation process for feature selection, hyperparameter selection, and model evaluation. In each repetition, data is split into 5 folds (1–5). In each fold, 80% are used in an internal CV process for feature selection, hyperparameter optimization, and model training (green squares). The resulting model is tested on the remaining 20% (red square), recording the probability scores and the SHAP values of the random forest model. CV: Cross-validation. SMOTE: Synthetic minority oversampling technique. SHAP: Shapley additive explanations.

Subsequently, repeated nested k-fold stratified cross-validation (CV) was performed [25], employing an outer CV loop for model evaluation and an inner CV loop for feature and model selection (**Fig 1**). In each step of the outer CV loop (k = 5, value chosen for practicability), the values of each feature vector in the training set were standardized and passed on to the inner CV loop (k = 4, chosen for practicability). To account for class imbalance, artificial instances of the minority class were created by using the synthetic minority over-sampling technique (SMOTE) on the training data [26]. During each step of the inner CV, Boruta feature selection was performed [27,28]. Feature selection frequencies were recorded as a measure of selection stability [29]. Following a voting strategy [30], features selected in more than one fold of the inner CV were used as the final feature set. Hyperparameters were chosen to maximize the area under the ROC curve (AUC). A random forest classifier was trained on the whole outer training set with the hyperparameter and feature set found during the inner CV, and evaluated on the outer test set. Additionally, a logistic regression classifier and a support vector machine classifier (both following the implementation of the Python package *sklearn*) were tested.

Feature importance was assessed by calculating Shapley additive explanations (SHAP) values from the fitted random forest classifier [31]. To account for the influence of differences in CV splits, the process was repeated 50 times with different pseudorandom number generator initiation seeds.

### Statistical analysis

Continuous variables are expressed as mean ± standard deviation and 95% confidence interval (CI), categorical variables as count or percentage. Inter- and intrareader agreement on radiomic features was assessed by calculating the intraclass correlation coefficient for consistency in a two-way mixed approach (ICC(3,1)). Selected features were plotted for visual analysis, tested for normality using the Shapiro-Wilk test and compared using two-tailed t-tests or Mann-Whitney-U tests as appropriate. Pearson’s correlation coefficient *r* was calculated for each feature pair and the results were visually analyzed. Proportions of categorical variables were tested using a chi-squared test for independence. A p-value below 0.05 was regarded as indicative of statistical significance. Inter-rater agreement for radiologic evaluation of tumor stage and risk group was assessed by calculating Cohen’s kappa. Kappa values were interpreted as indicating (almost) perfect (>0.9), strong (<0.9–0.8), moderate (<0.8–0.6), weak (<0.6–0.4), minimal (<0.4–0.2) or no agreement (<0.2). Radiologists’ performance is expressed as sensitivity and specificity. Model performance was calculated from the out-of-fold predictions, averaged across repetitions and is expressed as area under the ROC curve (AUC), and accuracy, sensitivity, specificity and F1 score (using the threshold determined by Youden’s index). 95% confidence intervals (CI) for each statistic were computed by bias-corrected bootstrapping. AUC values were interpreted as excellent (AUC 1.0–0.9), good (<0.9–0.8), fair (<0.8–0.7), poor (<0.7–0.6), not discriminating (<0.6). Statistical analysis was performed using Python (version 3.7.0) with libraries *scipy* (version 1.3.1) [32], *scikit-learn* (version 0.22.2) [33], *imbalanced-learn* (version 0.6.2) [34] and *shap* (version 0.37.0) [35].

## Results

### Patient characteristics

Of 105 identified patients undergoing surgery for TETs between the years 2000 and 2018, 43 patients had to be excluded for lack of compatible imaging (n = 33), for lack of a contrast-enhanced CT study (n = 7), or for the presence or previous treatment of another tumor (n = 3). Patient characteristics are summarized in **Table 1**. There was no statistically significant difference between LRT and HRT, between early and advanced TETs, between cases with and without myasthenia gravis, or pericardial infiltration regarding gender (p > 0.05) or age (p > 0.18).

**Table 1:** Patient characteristics.

		Risk (WHO)			Stage (TNM)			Myasthenia gravis		
		LRT	HRT		early	advanced		No	Yes	
		(n = 34)	(n = 28)	p-value	(n = 49)	(n = 13)	p-value	(n = 48)	(n = 14)	p-value
**Characteristic**										
Age (y), mean ± SD		58.8 ± 14.8	54.0 ± 12.4	0.182	57.5 ± 14.6	53.3 ± 10.8	0.334	57.4 ± 14.4	53.9 ± 12.3	0.410
Sex (n)				0.661			0.816			0.052
	female	14	14		23	5		18	10	
	male	20	14		26	8		30	4	
Diameter (mm), mean ± SD		71.8 ± 34.9	79.2 ± 32.7	0.130	71.0 ± 34.1	90.6 ± 29.1	0.011	78.6 ± 35.3	63.0 ± 25.7	0.062
**TNM Stage (IASLC/ITMIG)**				0.001			-			0.673
	I	32	17		49	0		37	12	
	II	0	0		0	0		0	0	
	III	2	2		0	4		3	1	
	IV	0	9		0	9		8	1	
**WHO Type**				-			<0.001			<0.001
	A	10	0		10	0		10	0	
	AB	16	0		16	0		15	1	
	B1	8	0		6	2		7	1	
	B2	0	13		11	2		5	8	
	B3	0	9		5	4		5	4	
	C	0	6		1	5		6	0	

IASLC: International Association for the Study of Lung Cancer. ITMIG: International Thymic Malignancy Interest Group. TNM: Tumor-node-metastasis. WHO: World Health Organization. SD: Standard deviation. P-values were derived from t-tests (age), Mann-Whitney U tests (diameter), or chi-squared tests for independence (categorical variables).

### Radiologists’ performance

The inter-reader agreement between radiologists was strong for the assessment of the tumor stage (early vs. advanced TET; κ = 0.86; percent agreement 93.5%). The sensitivity and specificity were 84.6% and 77.6% for reader 1 and 92.3% and 71.4% for reader 2, respectively.

Inter-reader agreement was weak for the differentiation of high-risk vs. low-risk lesions (κ = 0.58; percent agreement 79%). Radiologists reached a sensitivity and specificity of 53.6% and 50% for reader 1 and 75% and 41.2% for reader 2, respectively.

### Feature selection

**Fig 2:**
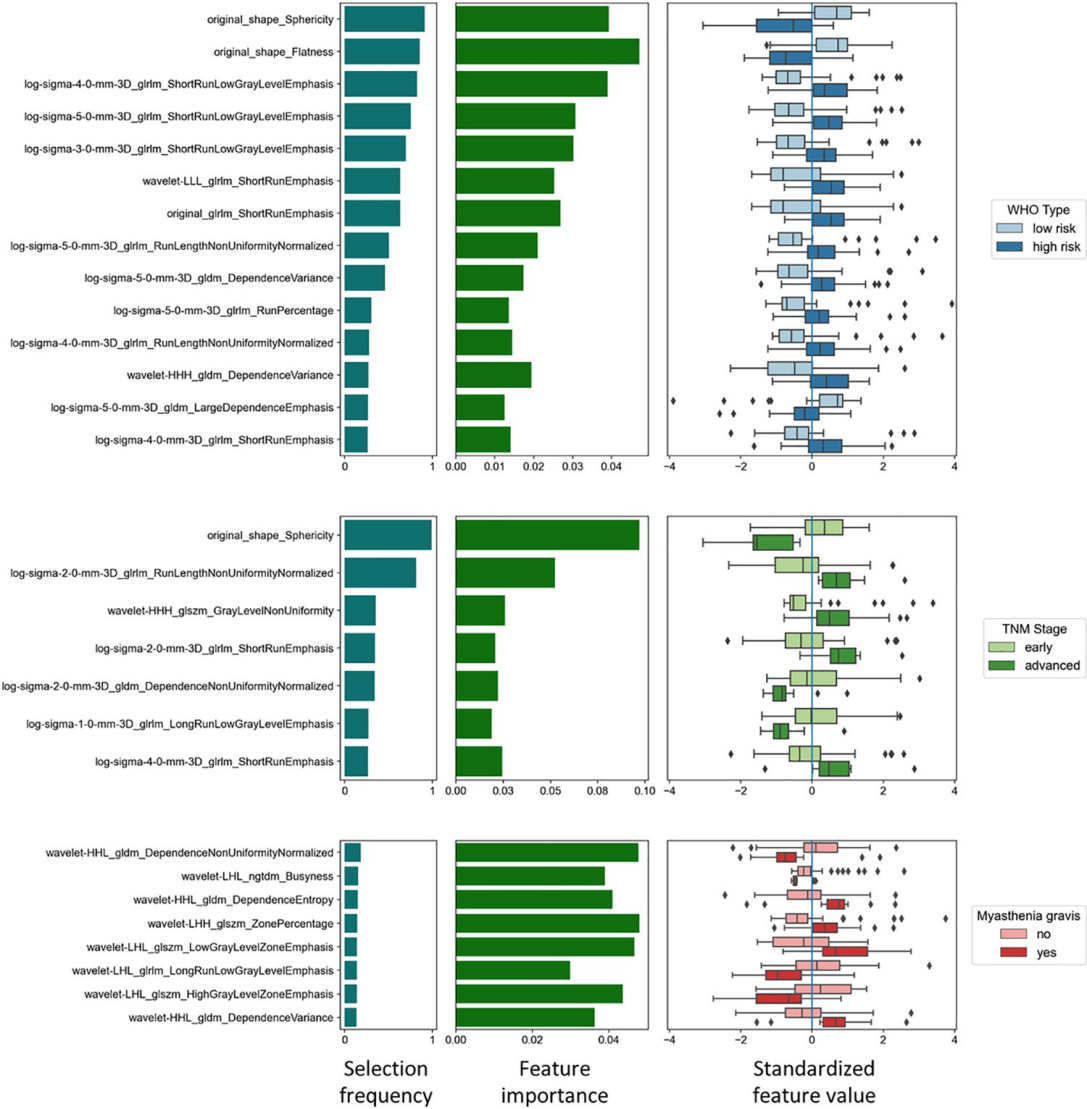
Feature selection. Features selected for the prediction of **a)** histologic subtype (WHO classification, low-risk vs. high-risk tumors), **b)** TNM stage (IASLC/ITMIG, early vs. advanced stage) and the presence of **c)** myasthenia gravis. The bar plots on the left display how often features were selected across folds as an indicator of selection stability. The bar plots in the center show the feature importance measured by the mean absolute SHAP values, representing the impact of a feature on the individual model prediction. The boxplots on the right display the individual standardized feature values grouped by the underlying category. The selected feature values differed significantly for all tested categories (p<0.05).

**Fig 3:**
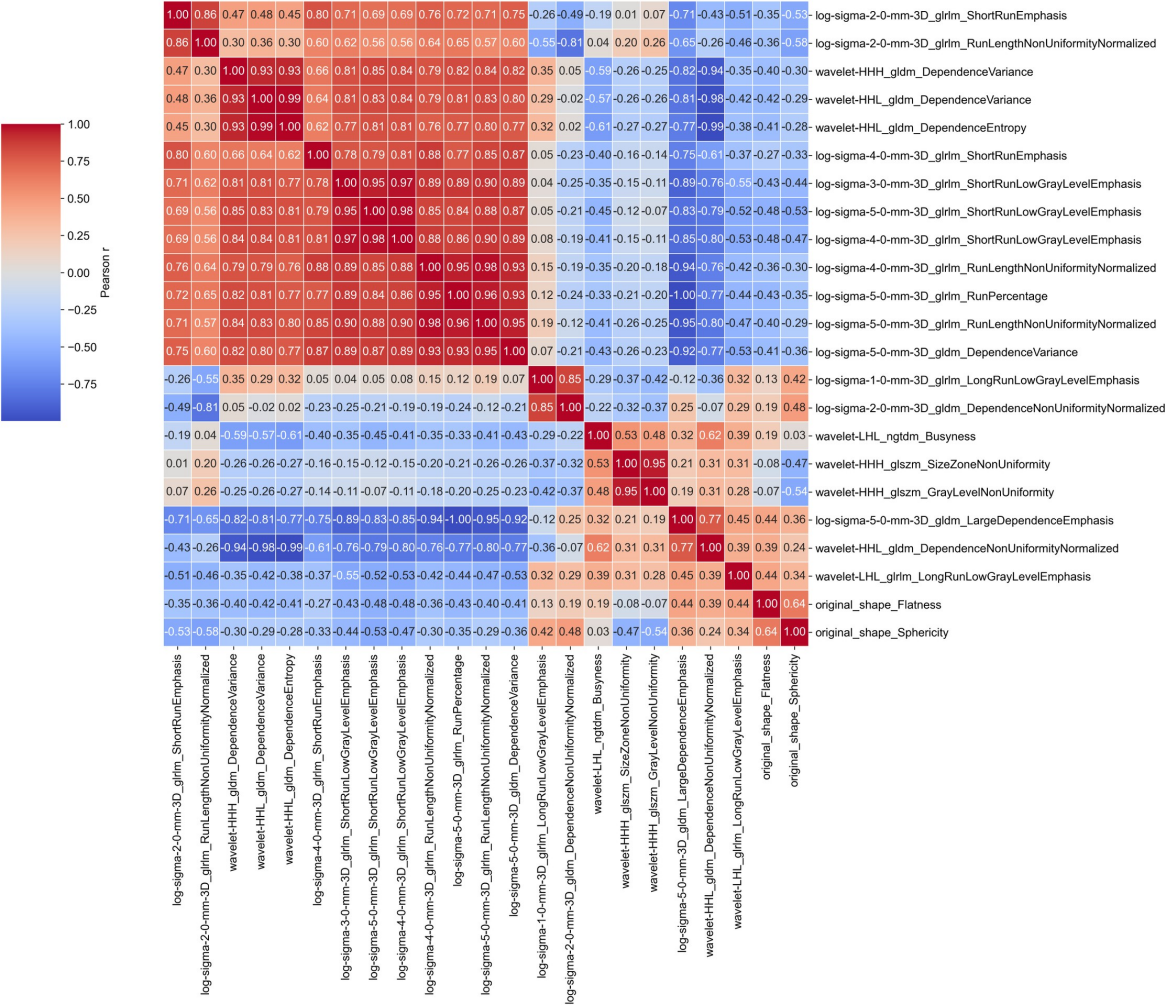
Correlogram of all selected radiomic features. Colors and numbers show Pearson’s correlation coefficient *r* of the respective feature pair.

334 of initially 1316 features were excluded after intra- and inter-reader analysis. The remaining features were used in the repeated nested cross-validation pipeline. Feature selection frequencies across folds were recorded as a measure for selection stability, and SHAP values were recorded to assess feature importance (**Fig 2**). Some of the selected features exhibited strong positive or negative correlations (**Figs 3 and** S1–S3).

**Fig 4:**
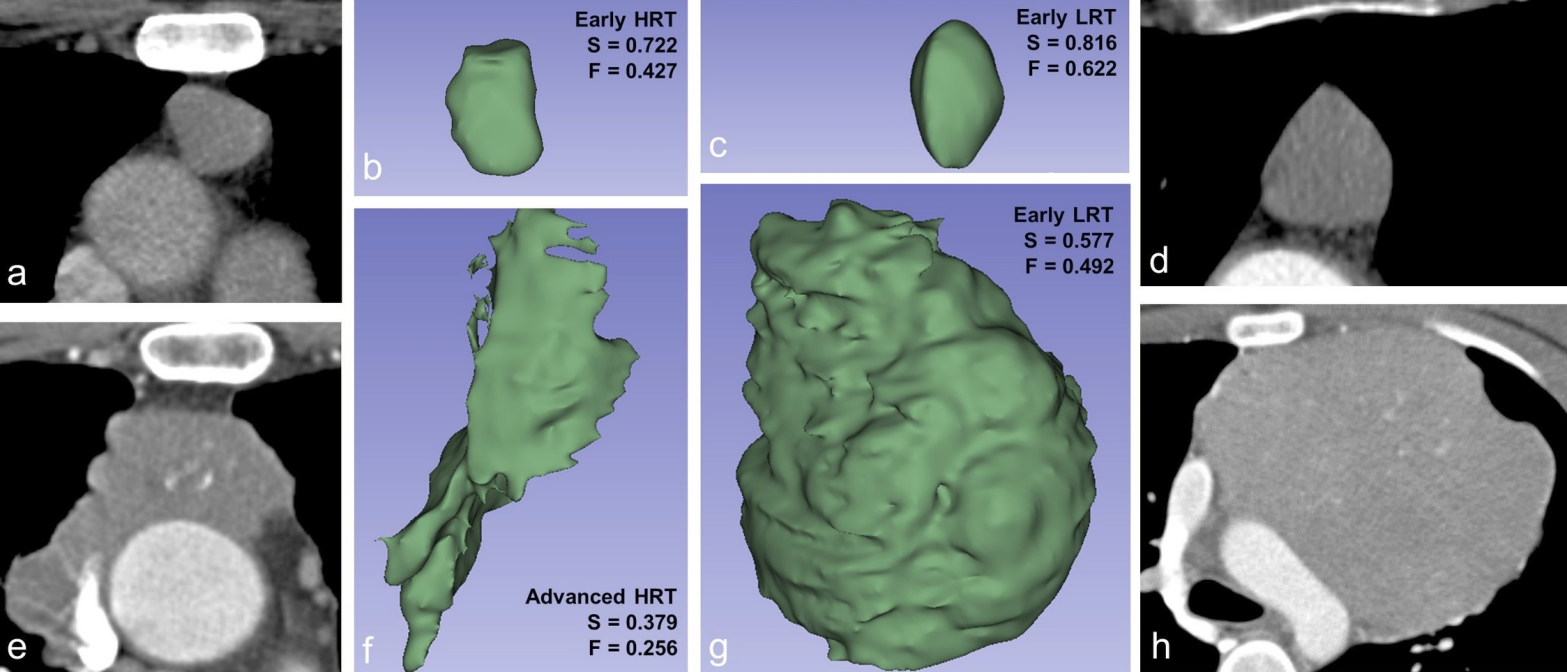
Axial CT slices (a, d, e, h) and corresponding volume renderings (b, c, f, g) of segmented VOIs with calculated values for sphericity (S) and flatness (F). LRT: Low-risk thymic epithelial tumor. HRT: High-risk thymic epithelial tumor.

Twelve features were identified for the classification of the histologic type into LRT or HRT (**Fig 2**A). The shape features *sphericity* and *flatness* (**Fig 4**) were frequently selected, significantly higher in LRT (sphericity, 0.72 ± 0.06 vs. 0.60 ± 0.08 in HRT; flatness 0.60 ± 0.12 in LRT vs. 0.43 ± 0.11 in HRT, p<0.001) and displayed high absolute SHAP values, suggesting a high impact on the prediction of the histology type. Among the texture features, the most important feature was the GLRLM feature *short run low gray level emphasis* (SRLGE) after applying the LoG filter (σ = 4, LRT 0.11 ± 0.04 vs. 0.14 ± 0.03 in HRT, p<0.001). The values of all selected features differed significantly between LRT and HRT (p<0.003).

Seven features were selected for the prediction of the TNM stage (**Fig 2**B), with all feature values differing significantly between early and advanced stages (p<0.003). The shape feature sphericity (advanced-stage TET, 0.47 ± 0.13 vs. early-stage TET, 0.54 ± 0.14, p<0.001) was most frequently selected and displayed the highest mean absolute SHAP value.

Using the criterion “selected at least once per CV fold” (threshold frequency 0.25), no features could consistently be selected for the prediction of myasthenia gravis. Experimentally lowering the threshold to 0.125 yielded five features (**Fig 2**C, p<0.017 for all comparisons).

### Model performance evaluation

**Fig 5:**
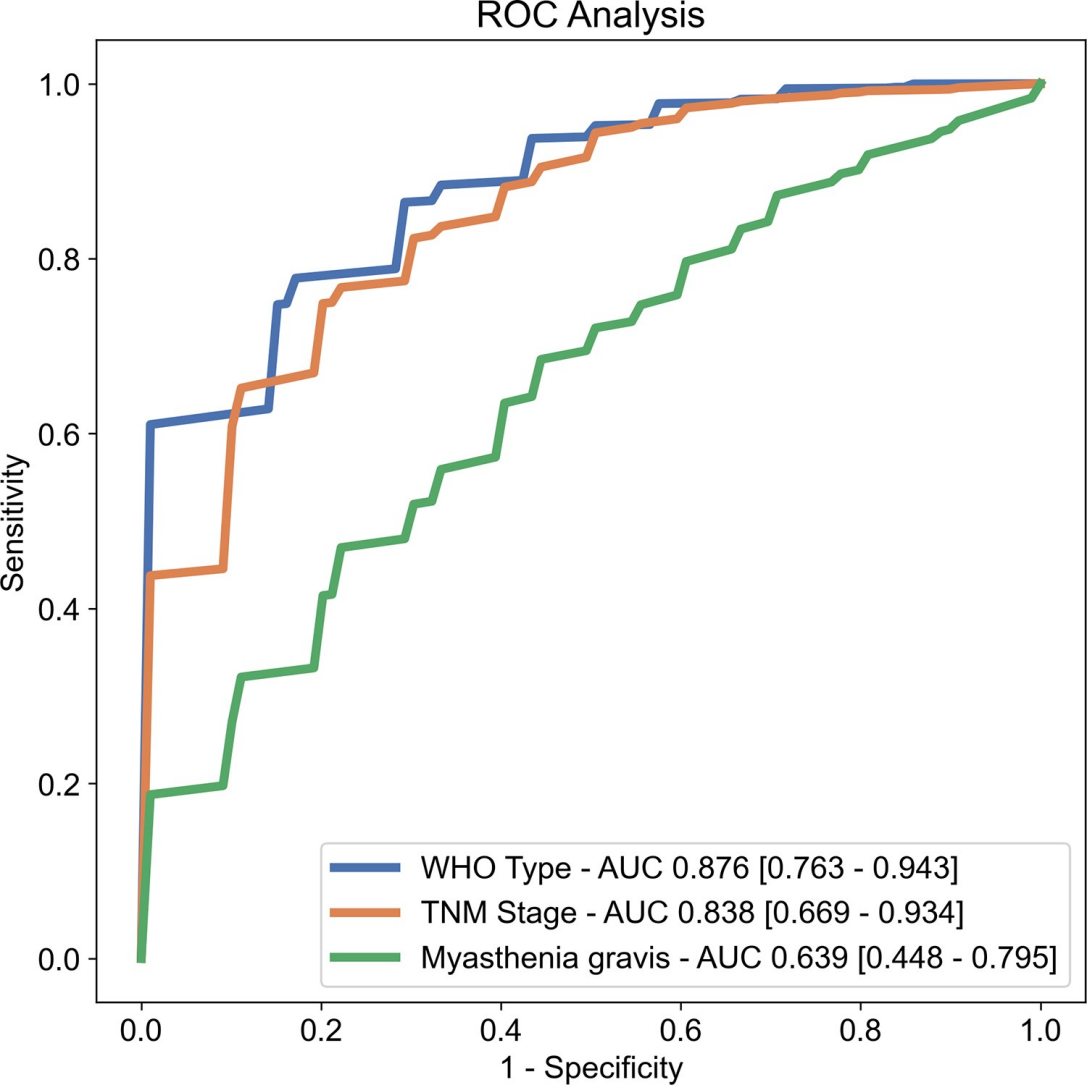
Receiver operator characteristic curves of the random forest classifier performance in each category. Values in square brackets indicate 95% confidence intervals. AUC: Area under the ROC curve.

**Fig 6:**
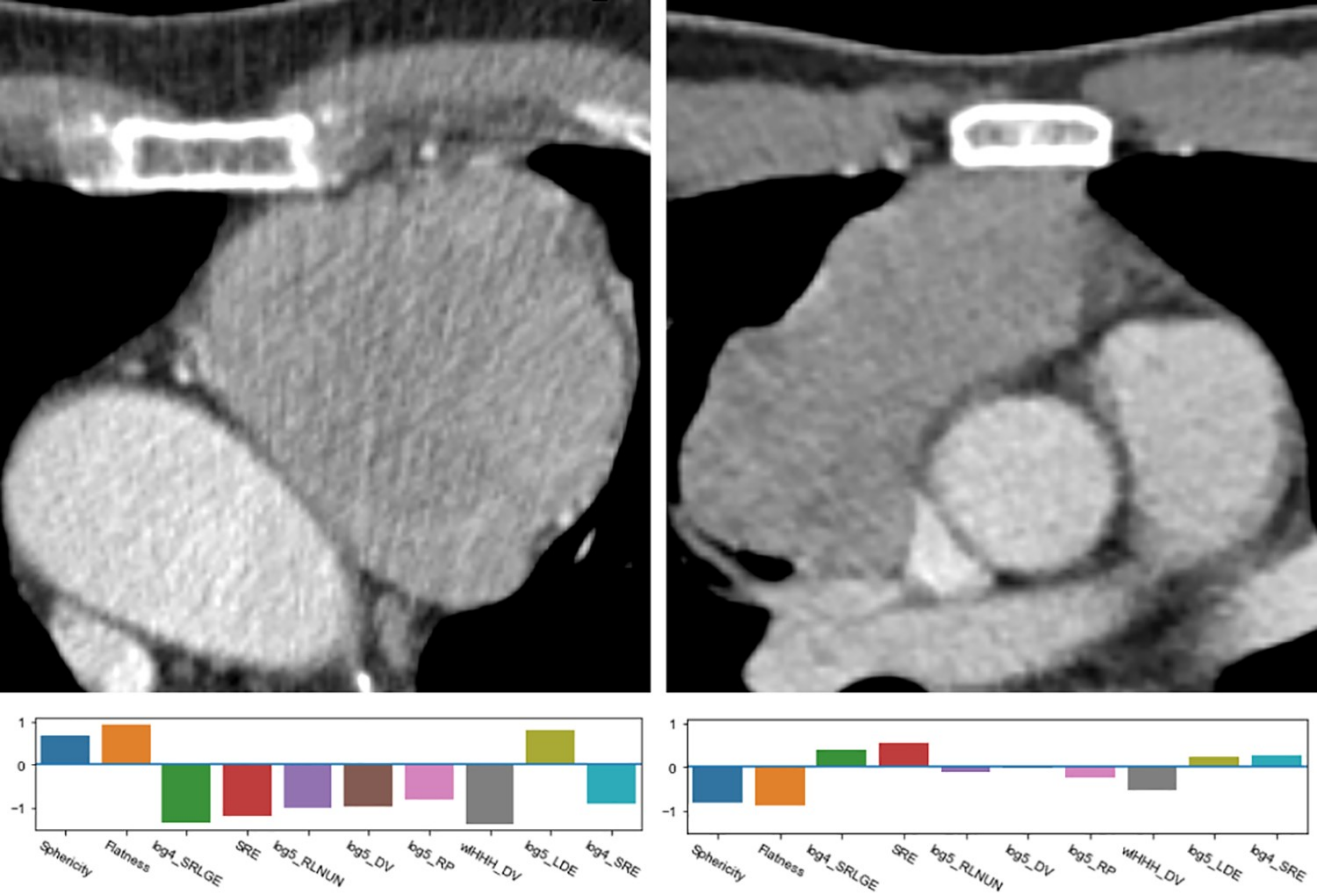
Comparison of a low-risk TET (LRT, left image) and high-risk TET (HRT, right image) with corresponding radiomic fingerprints. Both tumors have been captured in early stages. DV: Dependence variance. LoG*n*: Laplacian of Gaussian filter with σ = n. RLNUN: Run length non-uniformity. SRLGE: Short run low gray emphasis. SRE: Short run emphasis. wlHHH: Wavelet filter with high-pass filters (H) in every spatial direction. LDE: Large dependence emphasis.

Random forest classifiers were trained on the outer training sets and evaluated on the outer test sets (**Table 2**, **Fig 5**). The prediction of the histologic subtype (**Fig 6**) displayed the best performance (average ROC AUC, 0.876; 95%CI 0.763–0.943), followed by the tumor stage (average AUC, 0.838; CI, 0.669–0.934). Poor predictive performance was reached for the prediction of myasthenia gravis (average AUC, 0.639; CI, 0.448–0.795). Experimentally switching to a base estimator from a different classifier family did not significantly alter the discriminatory performance in any category (S1 Table).

**Table 2:** Random forest classifier performance.

Category	AUC (%, [CI])	Accuracy (%, [CI])	Sensitivity (%, [CI])	Specificity (%, [CI])	F-Measure (%, [CI])
**WHO Type**	87.6 [76.3–94.3]	77.5 [63.5–85.4]	77 [56.8–89.9]	77.8 [60.4–89.7]	75.5 [60.3–86.4]
**TNM Stage**	83.8 [66.9–93.4]	75 [60.8–83.4]	74.9 [40.6–93.6]	75.1 [60.8–85.7]	56 [33.4–74.1]
**Myasthenia gravis**	63.9 [44.8–79.5]	61.5 [47.3–71.6]	61.1 [30.4–83.9]	61.6 [46.9–74.5]	42.1 [23–61.3]

Performance metrics of the random forest classifier with 95% confidence intervals (CI, square brackets). AUC: Area under the receiver-operator characteristic curve.

## Discussion

This retrospective study evaluated the utility of CT-derived radiomics for the prediction of factors relevant to the prognosis and therapy selection in patients with TET. The discriminatory performance was good for predicting histologic subtype and TNM stage, and poor for the detection of myasthenia gravis.

Previous studies using CT-based radiomics for the prediction of the tumor stage [13,19] mainly used the well-established Masaoka-Koga staging system, which was created based on a small cohort of patients [20]. Recommendations from the International Association for the Study of Lung Cancer (IASLC) and the International Thymic Malignancy Interest Group (ITMIG) have been integrated into the 8^th^ edition of the TNM classification. TET with infiltration of well resectable structures (e.g. mediastinal pleura, pericardium) have been moved to TNM stages I and II, while the same TET were previously included in the highly heterogeneous stage III defined by the Masaoka-Koga classification, alongside TET infiltrating structures difficult to resect (e.g. great vessels, myocardium) [36]. The TNM staging system is thus considered to be more appropriate for TET evaluation by helping to formalize resectability [8].

In a recent study, an elastic net penalized logistic regression model created from CT-based radiomics exhibited fair discriminatory performance (AUC 0.708) to differentiate early from advanced TNM stage TET [18]. Possible limitations of this approach are given if there are non-linear relationships between features and the logit of the outcome variable, or complex interactions among the features, which might be overcome by other classifiers. For instance, a support vector machine trained on MRI-based radiomic features derived from multiple sequences reached an AUC of 0.88 for the same task [21]. To the best of our knowledge, our study is the first to use machine learning classifiers trained on CT-based radiomics to predict the TNM stage of TET. The RF classifier exhibited good discriminatory performance (AUC 0.838). While the specificity of the RF was similar, both radiologists exhibited higher sensitivity for detecting advanced stage lesions.

The histologic subtype bears implications for therapy selection. Low-risk TET (LRT) can often be resected completely, and less frequently require additional radio-/chemotherapy [37]. Estimating the histologic category based on visual features as heterogeneous enhancement, irregular margins, and necrotic components showed weak interreader agreement and resulted in poor performance. This is in line with the findings of Yasaka et al., who reported a poor discriminatory performance reached by visual assessment of TET heterogeneity (AUC 0.46–0.50) [12]. In contrast, the RF classifier in our study was able to distinguish LRT from high-risk TET (HRT) with good discriminatory performance (AUC 0.88). The performance was similar to the findings of Hu et al., who previously explored the use of CT-based radiomics derived from unenhanced and contrast-enhanced scans and machine learning classifiers for the same task and reached an AUC of 0.81 (RF classifier) [15], as well as to the findings of Ren et al., who used logistic regression to build a nomogram (AUC 0.86) [16]. Our study therefore supports the thought that quantitative, radiomic assessment of the tumor composition may outperform visual assessment of heterogeneity and shape and may enrich TET diagnostics. While this study relied on automatically extracted CT-based features alone, adding non-radiomic imaging features might further improve classifier performance: for instance, a study by Nakajo et al. reported an excellent AUC of 0.99, using both CT-based radiomics and maximum standardized uptake value from ^18^F-FDG PET/CT scans [38]. Shen et al. reached good discriminatory performance (ROC AUC 0.84) by integrating the TNM stage and a radiomics-based score in a nomogram model for the differentiation of low vs. high-risk TET [17].

In our study, the predictive performance of the RF-based model for MG was poor (AUC 0.64), and the selected feature sets were less consistent than for the other categories. A possible explanation might be the limited amount of available training data. For the same task, Liu et al. assembled a cohort of 230 TET patients and reported the creation of a deep neural network with a fair discriminatory performance (AUC 0.76), outperforming different machine learning classifiers trained on CT-based radiomic features (AUC 0.70–0.75, validation cohort) [39].

Interestingly, the shape feature sphericity appeared in multiple previous studies, using different means of feature selection, as an important feature for TET characterization [13,16,21,40,41]. In our study, sphericity was among the most important features for predictions of the histologic subtype as well as the TNM stage. Sphericity is the ratio of the surface area of a sphere with the same volume as a given VOI to the surface area of the VOI and was higher in low-risk and early-stage TET. Sphericity has also been described as a discriminating factor in other oncologic radiomic studies, e.g. in prostate cancer outcome [42].

Selected texture features predominantly stemmed from the gray level run length matrix (GLRLM) and gray level size zone matrix (GLSZM). For instance, significantly lower values of the features *short run low gray level emphasis* (SRLGE) and *run length non-uniformity normalized* (RLNN) in LRT indicated a higher homogeneity than in HRT, and low values of *size zone non-uniformity* (SZN) in early-stage TET indicated a higher homogeneity among the size zones than in advanced-stage TET. These findings are in line with previous findings connecting tumor heterogeneity with the potential of malignancy [2].

The dataset showed considerable heterogeneity in the CT scanner models as well as in the employed scanning parameters (e.g. tube voltage, slice thickness, kernel), which is known to influence the values of radiomic features [43,44]. This setup is a “real world” example reflective of the high variability in CT scanner models, scan and reconstruction parameters across institutions. Elaborate techniques like ComBat can compensate for technical differences in CT radiomics data but require more samples per subgroup than were available in our study [45]. Shape features were more consistently selected than texture features, supporting the knowledge that some features (e.g. spatial relationships) are more likely to be preserved across different acquisition techniques than others [46].

An advantage of the pursued approach was that, apart from the segmentation step, the pipeline introduced in this paper is mainly data-driven, with features and hyperparameters being picked without human intervention. The Boruta algorithm does not account for multicollinearity and may produce redundant features, and indeed several features showed high correlation coefficients. However, this helps to identify all features “relevant to the subject of interest, instead of merely building a black box predictive model” [27]. Additionally, RF models are known to work well with highly correlated features.

Understanding the output of machine learning models is often non-trivial, and explainability is important considering the responsibility of medical decision-making. In our study, SHAP values helped to increase the explainability by assessing the impact of individual features on both individual predictions and across the whole model [31]. The partial overlap of the selected features in this study with feature sets described in previous studies adds to the confidence in the acquired results.

There were limitations to our study. The retrospective study design bears a risk of selection bias, and prior to clinical utilization, the value of radiomics in TET characterization needs to be validated in future prospective studies.

Secondly, despite a long inclusion range, only a comparably small number of samples could be included in the final study cohort, which impeded the creation of a data split with training, validation, and test set. In a future study, the feature sets established in this study could be validated on an external cohort.

Thirdly, the histopathologic report, which served as the gold standard for our study, was often created by one pathologist, while there might be inter-observer variability in the assessment of the histologic tumor type.

## Conclusion

CT-based radiomics may serve as imaging biomarkers for the prediction of TET histology and TNM stage. The shape feature sphericity exhibited discriminative value in both categories, confirming previous studies. Radiomic models could be further evaluated to serve as an additional decision criterion for selecting the surgical approach, or for patients with contraindications for biopsy and therefore contribute to the goal of personalized medicine.

## Supporting information

S1 Fig Correlogram of features selected for the differentiation of low-risk and high-risk TET. The numbers represent Pearson’s *r* coefficient for the respective feature pair.
(DOCX)

S2 Fig Correlogram of features selected for the differentiation of early and advanced TNM stage TET. The numbers represent Pearson’s *r* coefficient for the respective feature pair.
(DOCX)

S3 Fig Correlogram of features selected for the prediction of myasthenia gravis. The numbers represent Pearson’s *r* coefficient for the respective feature pair.
(DOCX)

S1 Table Performance of different methods for the three studied classification tasks. AUC: Area under the ROC curve. CI: Confidence interval. RF: Random forest classifier. LR: Logistic regression classifier. SVM: support vector machine classifier.
(DOCX)

S2 Table TET radiomic features. (ZIP)

## Abbreviations

CV: Cross-validation
DICOM: Digital Imaging and Communications in Medicine
GLCM: Gray Level Co-occurrence Matrix
GLDM: Gray Level Dependence Matrix
GLRLM: Gray Level Run Length Matrix
GLSZM: Gray Level Size Zone Matrix
HRT: High-risk thymic epithelial tumor
ICC: Intraclass correlation coefficient
LoG: Laplacian of Gaussian
LRT: Low-risk thymic epithelial tumor
MRI: Magnetic resonance imaging
NGTDM: Neighboring Gray Tone Difference Matrix
NifTI: Neuroimaging Informatics Technology Initiative
PACS: Picture archiving and communication system
RF: Random forest (classifier)
ROC: Receiver operating characteristic
SHAP: SHapley Additive exPlanations
SMOTE: Synthetic minority over-sampling technique
TET: Thymic epithelial tumor
VOI: Volume of interest
