# Supplementary material for: Computed tomography radiomics for the prediction of thymic epithelial tumor histology, TNM stage and myasthenia gravis
Source: PLoS One. 2021 Dec 20;16(12):e0261401. doi: 10.1371/journal.pone.0261401 (PMC8687592; doi:10.1371/journal.pone.0261401)
Supplement: S2 Fig — The numbers represent Pearson’s r coefficient for the respective feature pair. (DOCX) [file pone.0261401.s002.docx]

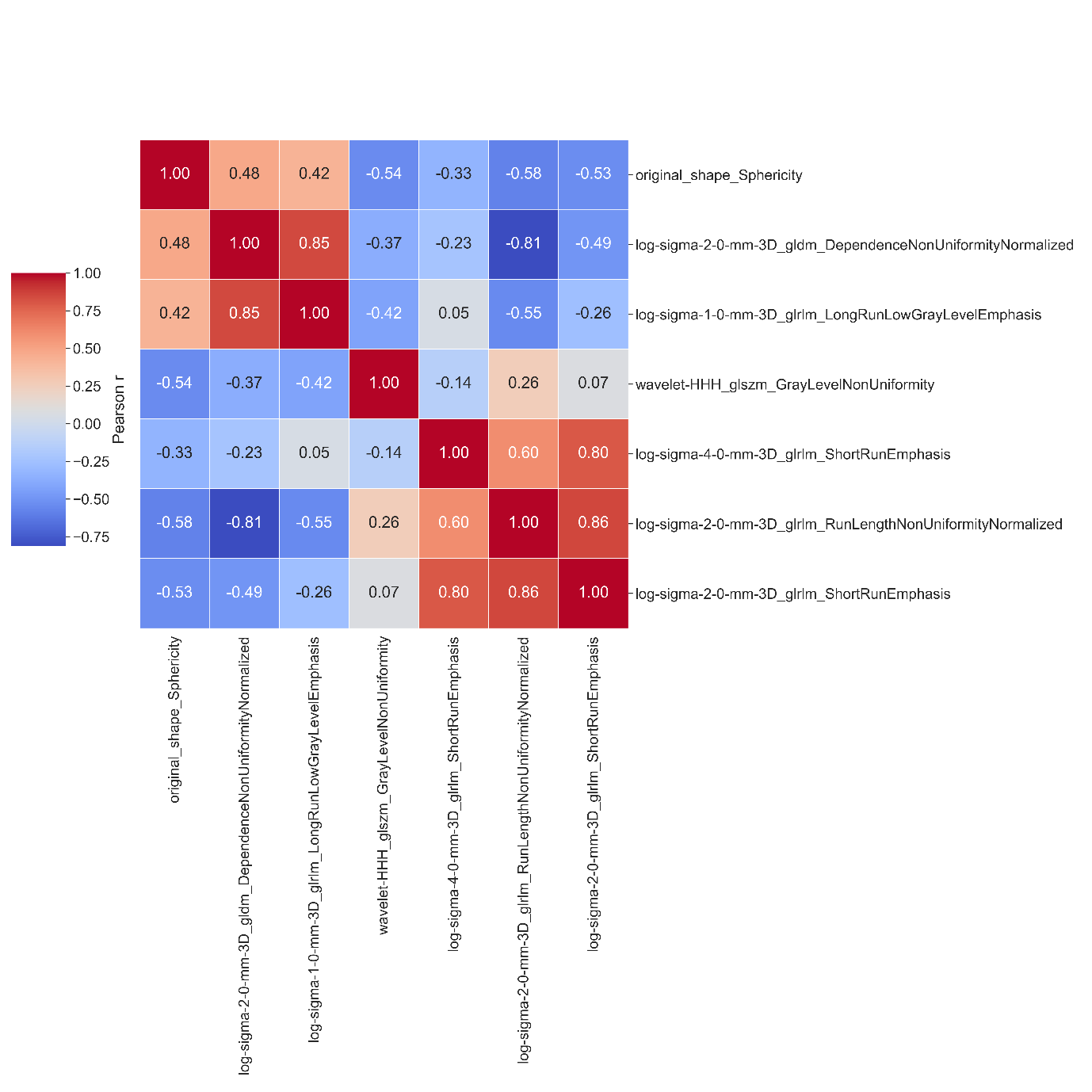


**Supplementary Figure S2**: Correlogram of features selected for the differentiation of early and advanced TNM stage TET. The numbers represent Pearson’s *r* coefficient for the respective feature pair.
