# Supplementary material for: Computed tomography radiomics for the prediction of thymic epithelial tumor histology, TNM stage and myasthenia gravis
Source: PLoS One. 2021 Dec 20;16(12):e0261401. doi: 10.1371/journal.pone.0261401 (PMC8687592; doi:10.1371/journal.pone.0261401)
Supplement: S1 Table — AUC: Area under the ROC curve. CI: Confidence interval. RF: Random forest classifier. LR: Logistic regression classifier. SVM: support vector machine classifier. (DOCX) [file pone.0261401.s004.docx]

**Supplementary Table S1**: Performance of different methods for the three studied classification tasks. AUC: Area under the ROC curve. CI: Confidence interval. RF: Random forest classifier. LR: Logistic regression classifier. SVM: support vector machine classifier.

|  | **AUC**  (%, [CI]) | **Accuracy**  (%, [CI]) | **Sensitivity**  (%, [CI]) | **Specificity**  (%, [CI]) | **F-Measure**  (%, [CI]) |
| --- | --- | --- | --- | --- | --- |
| **WHO Type** | | | | | |
| RF | 87.6 [76.3 - 94.3] | 77.5 [63.5 - 85.4] | 77 [56.8 - 89.9] | 77.8 [60.4 - 89.7] | 75.5 [60.3 - 86.4] |
| LR | 87.2 [75.0 - 94.4] | 80.6 [66.9 - 87.5] | 80.9 [60.9 - 92.4] | 80.4 [63.6 - 91.3] | 79.0 [64.6 - 88.7] |
| SVM | 87.3 [75.7 - 94.0] | 77.5 [63.3 - 85.0] | 78.4 [59.1 - 90.9] | 76.8 [59.8 - 89.0] | 75.9 [60.7 - 86.3] |
| **TNM Stage** | | | | | |
| RF | 83.8 [66.9 - 93.4] | 75 [60.8 - 83.4] | 74.9 [40.6 - 93.6] | 75.1 [60.8 - 85.7] | 56 [33.4 - 74.1] |
| LR | 86.6 [72.1 - 94.4] | 77.8 [63.8 - 85.5] | 78 [44.7 - 95.0] | 77.8 [64.1 - 87.8] | 60.1 [38 - 77.6] |
| SVM | 83.6 [64.1 - 93.8] | 76.4 [62.3 - 84.3] | 75.8 [40.8 - 93.7] | 76.5 [62.7 - 86.6] | 58.1 [35.9 - 76.2] |
| **Myasthenia gravis** | | | | | |
| RF | 63.9 [44.8 - 79.5] | 61.5 [47.3 - 71.6] | 61.1 [30.4 - 83.9] | 61.6 [46.9 - 74.5] | 42.1 [23 - 61.3] |
| LR | 56.2 [36.0 - 75.0] | 56.7 [42.3 - 67.0] | 56.8 [26.3 - 80.6] | 56.7 [42.2 - 69.8] | 37.7 [19.6 - 56.8] |
| SVM | 63.7 [43.5 - 80.0] ) | 58.0 [43.6 - 68.3] | 59.6 [29.1 - 82.9] | 57.5 [42.5 - 70.4] | 39.3 [21.1 - 58.2] |
